# An Enhanced Hybrid Astar Path Planning Algorithm Using Guided Search and Corridor Constraints

**DOI:** 10.3390/s26020379

**Published:** 2026-01-07

**Authors:** Na Che, Xianwei Zeng, Jian Zhao, Haiyan Wang, Qinsheng Du

**Affiliations:** 1 College of Computer Science and Technology, Changchun University, Changchun 130022, China; zengxianwei2023@163.com (X.Z.); zhaojian@ccu.edu.cn (J.Z.); wanghy80@ccu.edu.cn (H.W.); duqs@ccu.edu.cn (Q.D.); 2 Ministry of Education Key Laboratory of Intelligent Rehabilitation and Barrier-Free Access for the Disabled, Changchun 130022, China; 3 Jilin Provincial Key Laboratory of Human Health State Identification and Function Enhancement, Changchun 130022, China

**Keywords:** path planning, HybridA* autonomous vehicles, guided search, safe corridor

## Abstract

Aiming at the problems of large search space, unstable computational efficiency, and lack of safety of generated paths in complex environments of traditional HybridA* algorithms, this paper proposes an improved HybridA* algorithm based on Voronoi diagrams and safe corridors (GCHybridA*) to overcome these challenges. The method first reduces ineffective node expansion by constructing a Voronoi path away from obstacles and smoothing it, followed by selecting key guidance points to provide stage-like goals for path search. Then, an innovative safe corridor is generated and the path search is restricted to the safe corridor area to guarantee the safety of the path, and an adaptive step-size mechanism is designed to balance the search efficiency and path quality. The experimental results show that the GCHybridA* algorithm significantly outperforms the conventional HybridA* algorithm, with an average reduction of 83.7% in node expansions while maintaining zero potential collision points across all four typical maps. This study provides an innovative and robust solution for efficient and safe path planning in autonomous driving systems. This study provides an innovative and robust solution for global path planning in autonomous driving systems, focusing on static environment navigation with safety guarantees.

## 1. Introduction

In the context of the rapid development of autonomous driving technology, path planning, as a core component of autonomous navigation, is crucial for ensuring safe, efficient, and comfortable vehicle travel. Typically, path planning is divided into two hierarchical layers: global path planning and local path planning.

Global path planning operates on a prior static environmental representation (e.g., a high-definition map) and is responsible for generating a long-term, coarse reference route from the start to the goal. Its key objectives are to ensure kinematic feasibility, maintain a safe distance from known static obstacles, and achieve high computational efficiency.

Local path planning, in contrast, relies on real-time sensor data to perform short-term, reactive trajectory refinement. It focuses on dynamic obstacle avoidance, precise path tracking, and compliance with traffic rules, all while adhering to the corridor provided by the global plan.

The two layers are intrinsically linked: global path planning establishes the topological and geometric corridor within which local planning operates, thereby providing the essential spatial and behavioral prior for real-time decision-making.

This work focuses specifically on the global path planning layer. Within this scope, we aim to develop algorithms that not only generate collision-free and kinematically feasible paths in static environments but also optimally balance computational complexity with path quality—a trade-off that is fundamental to deploying reliable autonomous systems in complex, unstructured scenarios.

The global path planning algorithms that are widely used so far are sample-based algorithms and graph-based algorithms. The RRT is an active sampling-based algorithm proposed by LaValle in 1998. The method is suitable for solving path planning problems with different motion constraints and has the ability to handle multi-degree of-freedom problems. The principle of the RRT algorithm is to quickly search the bit-shaped space nodes to generate a path connecting the starting and target nodes [1]. The PRM proposed by Overmars et al. in the early 1990s. This method is based on the sampling algorithm, which solves the difficulty of constructing an effective path graph in high dimensional space. This algorithm represents the connectivity of path graph by sampling in configuration space, collision detection of sampling points, and testing whether adjacent sampling points can be connected [2].

Yang Li et al. proposed a new adaptive step size RRT algorithm. By establishing norm compatible inequality of configuration space and workspace, the step size in workspace is constrained within the allowable range, and then effective collision detection is realized. The adaptive step size RRT method can determine the step size without multiple debugging, which improves the robot’s path planning speed [3]. Zhuohua Yang et al. proposed the LT-RRT* algorithm, which used line segment theorem to improve the selection of new nodes and surrounding parent nodes, and to some extent reduced path break points and improved the smoothness of the path [4]. Yangjie Li et al. proposed a new algorithm PQ-RRT*, which combined the advantages of P-RRT* (potential functions based RRT*) and Quick-RRT*. PQ-RRT* changes the sampling strategy by adopting the attraction of the target region and expands the search range of the selected parent node [5].

Among the path planning algorithms based on graph search, A* [6], Martins et al. [7] designed an improved multi-objective A* (IMOA*) algorithm for path planning of mobile robots in large workspaces. Wang et al. [8] introduced road condition indicators in the evaluation function of the A* algorithm to reduce the road cost of the mobile robot while walking.

Many experts and scholars have conducted in-depth research on path smoothing techniques [9], aiming at generating feasible driving trajectories for self-driving vehicles [10,11,12], and in the course of their research, they have also given full consideration to obstacle avoidance to ensure that the vehicles drive in a safe environment [13]. However, path smoothing methods usually optimize or smooth this path after the search algorithm generates an initial path, which leads to the fact that path smoothing methods cannot actively evaluate or select other potential paths during the execution of the search algorithm, so this path smoothing approach lacks the ability to dynamically adjust or optimize the path during the search process.

The HybridA* algorithm is a hybrid path planning algorithm that combines graph search and sampling techniques, aiming at solving the path planning problem in complex environments considering motion constraints, and is especially suitable for vehicle path planning. However, the algorithm has a large search space and significant computational consumption, while the generated paths are usually too close to obstacles, posing a safety hazard. Therefore, the algorithm still needs further optimization and improvement in terms of improving safety and computational efficiency [14].

Numerous scholars have conducted in-depth research and improvement of the HybridA* algorithm. Tang et al. [15] introduced the repulsive force and attractive force in the artificial potential field method to optimize the algorithm for the problem that the output paths of the algorithm often contain unnecessary steering and are close to the obstacles, making the paths improve in both smoothness and safety. However, this method is applied as a post-smoothing technique to the HybridA* generated paths may miss the better paths during the search process. Chi et al. [16] reduced the search time and improved the efficiency of the algorithm by changing the computation of the HybridA* heuristic function. However, they did not explore the problem of large search space in depth. Dang et al. [17] improved the use of RS curves in the HybridA* algorithm by generating multiple RS curves with different curvature values and introducing a cost function to evaluate the choices, which solved the problem of possible collisions of RS curves in the vicinity of obstacles, especially at corners. However, their study on the safety of the forward expansion phase is still insufficient. Chang et al. [18] addressed the problem of the HybridA* algorithm generating ineffective searches in U-type obstacles by proposing a convex packet expansion to fill this type of obstacles, and experiments proved that this approach improved the algorithm’s search efficiency in this type of maps. Meng et al. [19] introduced an augmentation of the HybridA* algorithm, they integrated the Voronoi potential field into the cost function in terms of safety enhancement to keep the planned path away from obstacles, but did not utilize the Voronoi diagram to solve the computationally efficient problem. Lian et al. [20] guided the HybridA* algorithm in searching for an automated valet parking path by incrementally expanding a generating rectangle corridor as a way of enhancing the path’s safety.

Sedighi et al. [21] utilized the visibility graph to search for initial paths and set path points at corners to guide the HybridA* algorithm’s search process, which reduces the number of searches and improves efficiency. Sedighi et al. [22] proposed an improved HybridA* algorithm, which optimizes the path safety and computational efficiency by generating the guided paths through Voronoi diagrams and designing the potential field function, and experiments show that the method reduces the computational time consumption by 30% on average compared with the traditional HybridA*. However, there are some shortcomings in this method: first, the strategy of planning the path along the obstacles on one side in the open area is difficult to adapt to the unstructured and complex terrain; second, the real-time computation of the Voronoi potential field in the process of node expansion increases the computational complexity and cannot be pre-processed; third, the simple mechanism of generating the guidance points leads to a lack of representativeness of the generation of the guidance points in the complex environment, which affects the quality of the generated paths; Fourth, the oscillation problem arising from the difficulty of matching the nodes with the target points is not discussed, and these shortcomings limit the effectiveness of the algorithm in the application of complex environments. Cui et al. [23] proposed an automatic parking path planning method combining HybridA* algorithm and geometric curves and optimized the path using the idea of genetic algorithm, aiming to improve the efficiency and safety of the automatic parking system in a narrow space. Qin et al. [24] firstly searched for the initial path using the JPS algorithm, and then selected the guiding points on the path as the guiding points of the HybridA* algorithm, which effectively reduced the number of node extensions and improved the efficiency of the algorithm. However, their generated paths are too close to obstacles and have potential collision risks.

This paper proposes the GCHybridA* algorithm, which overcomes the deficiencies of the existing methods through the following innovative designs: firstly, the Voronoi path is smoothed and the key guidance points are extracted based on the sampling and screening mechanism, and at the same time, the arrival area is expanded for each guidance point; secondly, the Voronoi path is used to construct the safe corridor and divide the area, which is used to limit the search space and implement the dynamic step-size mechanism. The method reduces the ineffective expansion of the nodes and the redundant expansion near the target point, which fully guarantees the safety of the path, improves the adaptability of the environment and increases the robustness of the algorithm. It is especially suitable for safe and robust path planning tasks in complex dynamic environments.

The main contributions of this paper can be summarized as follows:

1. A novel forward guidance strategy based on smoothed Voronoi path extraction and adaptive arrival regions, which effectively reduces ineffective node expansions and provides stage-wise search objectives.
2. An innovative safe corridor construction method that dynamically constrains the search space and incorporates a step-size adaptation mechanism, ensuring path safety and enhancing search efficiency in complex environments.
3. The integration of guidance points and safe corridors into the HybridA framework, resulting in the proposed GCHybridA algorithm, which achieves a significant reduction in node expansions (83.7% on average) and eliminates potential collision points across all tested scenarios.

Comprehensive experimental validation is performed in four representative maps, demonstrating the algorithm’s robustness, computational stability, and superior performance in both safety and efficiency compared to state-of-the-art methods.

## 2. HybridA*

The HybridA* algorithm is an efficient algorithm for path planning of self-driving vehicles, which combines the heuristic search characteristics of the A* algorithm and the geometrically constrained characteristics of vehicle motion, thus ensuring that the generated paths not only fit the actual motion characteristics of the vehicle, which can also realize efficient path planning in complex environments.

**Figure 1:**
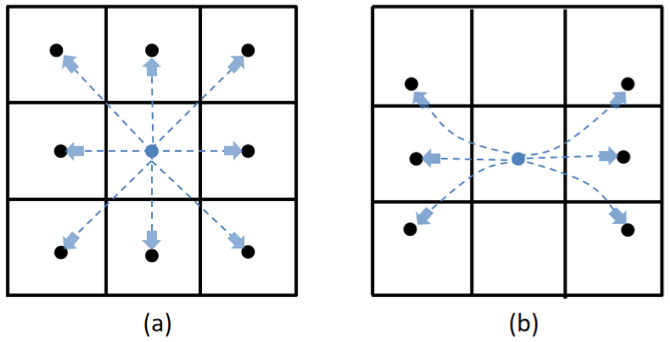
Comparison of node expansion methods: (**a**) A* algorithm; (**b**) HybridA* algorithm.

The core advantage of the HybridA* algorithm is its unique node expansion mechanism. Unlike the A* algorithm that simply expands to eight neighboring directions in a 2D grid environment to search for paths comprehensively, the HybridA* algorithm is based on the actual kinematic model of the vehicle and accurately computes a series of neighboring points that are both consistent with vehicle dynamics and practicable to execute, by taking into account the forward, backward, and cornering maneuvers. Figure 1 visualizes the comparison of these two extensions.

After performing the node extension step, the HybridA* algorithm evaluates the combined cost of each node based on Equation (1) and selects the node with the lowest current cost as the new parent node, thus incrementally extending towards the goal node to generate an optimal path.

(1) $$F\left(n\right)=w_{1}g\left(n\right)+min({w_{2}h}_{1},{w_{3}h}_{2})$$

where g(n) represents the known cumulative cost from the starting point to the current node; h_1_ is the heuristic cost when only the distribution of obstacles is considered and no vehicle kinematic constraints are involved; and h_2_ is the heuristic cost when only the vehicle kinematic constraints are considered and the presence of obstacles is ignored. w_1_, w_2_, and w_3_ are used as the weight coefficients of g, h_1_, and h_2_, respectively.

The HybridA* algorithm improves path planning by introducing realistic kinematic models and optimized cost function design, but the algorithm also faces two challenges.

**Figure 2:**
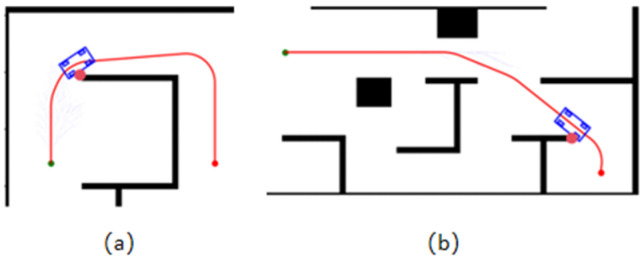
Schematic of collisions: (**a**) Collision generated by HybridA*; (**b**) Collision generated by RS curve. The small green dot is the starting point, and the small orange dot is the target point.

The HybridA* algorithm typically employs a two-stage strategy combining forward expansion with Reeds-Shepps curves when performing path planning. However, the paths derived from both planning stages run the risk of being too close to obstacles, as shown in Figure 2.

**Figure 3:**
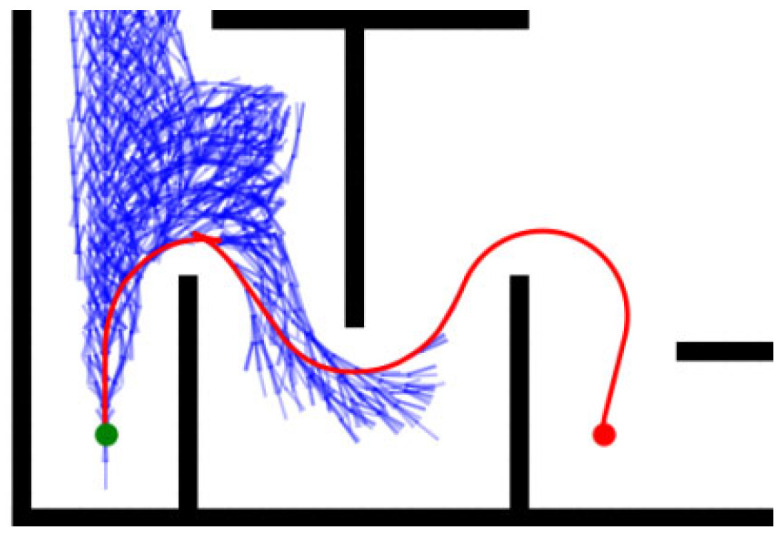
Schematic diagram of invalid extension. The green dot is the starting point, and the orange dot is the target point.

In addition, in order to balance the path quality, the heuristic cost of the algorithm is usually set to be limited, which leads to insufficient guiding information in the path search, triggering ineffective search and increasing the algorithm complexity and computational burden, as shown in Figure 3.

To address the above two challenges, this paper proposes a GCHybridA* algorithm that combines a forward guidance policy with a safe corridor policy, aiming to ensure that the search process maintains a stable low computational complexity while the generated paths always have excellent safety.

## 3. Forward Guidance Strategies

To address the above challenges, this study proposes an innovative forward guidance strategy that aims to improve the search efficiency and safety of the generated paths of the HybridA* algorithm by optimizing the arrival conditions and selecting key guidance points.

### 3.1. Voronoi Path Generation

In this study, we first construct a Voronoi graph based on a raster map, which is a technique of spatial segmentation based on the distribution of obstacles, for generating safe Voronoi edges away from surrounding obstacles. Then, we search for the shortest path from the starting point to the goal point and name it as Voronoi path, which serves as the basis for the subsequent stage-based search.

**Figure 4:**
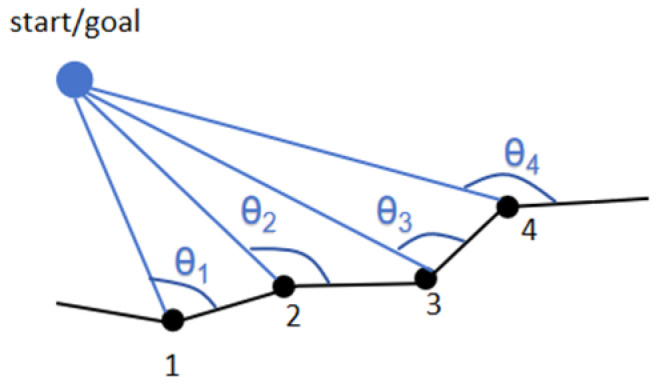
Schematic diagram of the first/end path smoothing approach.

Subsequently, we smooth the sharp corners in the Voronoi path to ensure the continuity and smoothness of the subsequent search process. Specifically, at the start and end points of the path, we calculate the angles between the line segments formed from the point to its four nearest Voronoi path points and the corresponding path edges (shown as θ1, θ2, θ3, θ4 in Figure 4), respectively. And, the Voronoi path point corresponding to the largest angle is selected to replace the original Voronoi point at the sharp corner (in Figure 4, i.e., point 4 is selected to replace point 1).

In the intermediate path segments, we use a smoothing algorithm based on the tangent circle. For each point P_1_ on the path, the angle between two adjacent edges formed by it and its front and back neighboring points P_0_ and P_2_ is calculated using Equation (2).

(2) $$\theta_{degrees}=\arccos\left(\frac{\vec{v_{0}}\cdot \vec{v_{1}}}{\left|\vec{v_{0}}\right|\left|\vec{v_{1}}\right|}\right)\times \frac{180}{\pi }$$

$\vec{v_{0}}$ and $\vec{v_{1}}$ are direction vectors pointing from P_1_ to P_0_ and P_2_, respectively. When θ_degrees_ is lower than the preset threshold θ_threshold_, it indicates that there is a large curvature change here, which needs to be smoothed by internal tangent circle.

**Figure 5:**
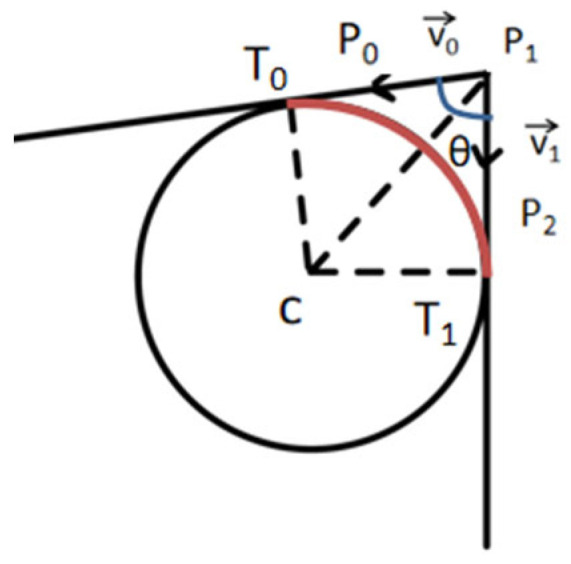
Schematic diagram of the calculation of circular arc segments.

The computational schematic is shown in Figure 5, and the unit vectors of the angular bisectors of $\vec{v_{0}}$ and $\vec{v_{1}}$ are calculated from Equation (3).

(3) $$\vec{bisector}=\frac{\frac{\vec{v_{0}}}{\left|\vec{v_{0}}\right|}+\frac{\vec{v_{1}}}{\left|\vec{v_{1}}\right|}}{\left|\frac{\vec{v_{0}}}{\left|\vec{v_{0}}\right|}+\frac{\vec{v_{1}}}{\left|\vec{v_{1}}\right|}\right|}$$

Then according to the sine theorem and the predefined radius r, the offset of the center of the circle relative to P_1_ can be calculated by Equation (4).

(4) $$d=\frac{r}{\sin\left(\frac{\theta }{2}\right)}$$

Then, based on the results obtained from Equations (3) and (4), the coordinates of the center of the circle C can be calculated by Equation (5).

(5) $$C=P_{1}+d\times \vec{bisector}$$

Denoting the perpendicular vector of $\vec{\nu_{0}}$ as $\vec{perp_{v}}0$ and the perpendicular vector of $\vec{v_{1}}$ as $\vec{perp_{v}}1$, the positions of the two tangent points of the internal tangent circle to the path are given by Equations (6) and (7).

(6) $$T_{0}=C+\vec{perp_{v}}0\times r$$

(7) $$T_{1}=C+\vec{perp_{v}}1\times r$$

Finally, the parametric Equations (8) and (9) generate the arc segment connecting these two tangent points and replace the original folded segment from T_0_ through P_1_ to the T_1_ portion.

(8) $$X\left(t\right)=C_{x}+r\cdot cos\left(t\right)$$

(9) $$Y\left(t\right)=C_{y}+r\cdot sin\left(t\right)$$

t is a variable between the start angle α and the end angle β, depending on the position of T_0_ and T_1_ with respect to C.

The smoothing arc radius r is a tunable parameter. Based on experimental analysis, this paper selects r = 3.0 m, a value that achieves a good balance between eliminating sharp corners in the path and preserving its original geometric shape.

**Figure 6:**
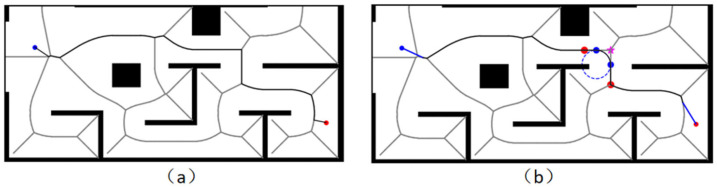
Comparison of Voronoi paths before and after smoothing: (**a**) shows the original Voronoi path; (**b**) shows the smoothed Voronoi path. The small blue dot is the starting point, and the small orange dot is the target point. The pentagram represents the corner point, while the large orange dot and large blue dot serve as auxiliary calculation points.

The initial generated Voronoi path is shown in Figure 6a, and the smoothed Voronoi path after the above method is shown in Figure 6b. The smoothed Voronoi path will be used as the basis for the next step of the guidance point selection.

### 3.2. Guidance Point Selection and Processing

The smoothed Voronoi path point set is sparsified at a predefined sampling rate, and then those points whose distances to obstacles are less than a predefined threshold are retained as guidance points, while the tangent line of the path at the guidance points is taken as their heading. Since the Voronoi path has a safety feature, the guide points selected based on the Voronoi path are also located far away from the surrounding obstacles, thus providing the algorithm with a safe stage goal to effectively guide the algorithm’s search direction and reduce the ineffective expansion during the search process.

**Figure 7:**
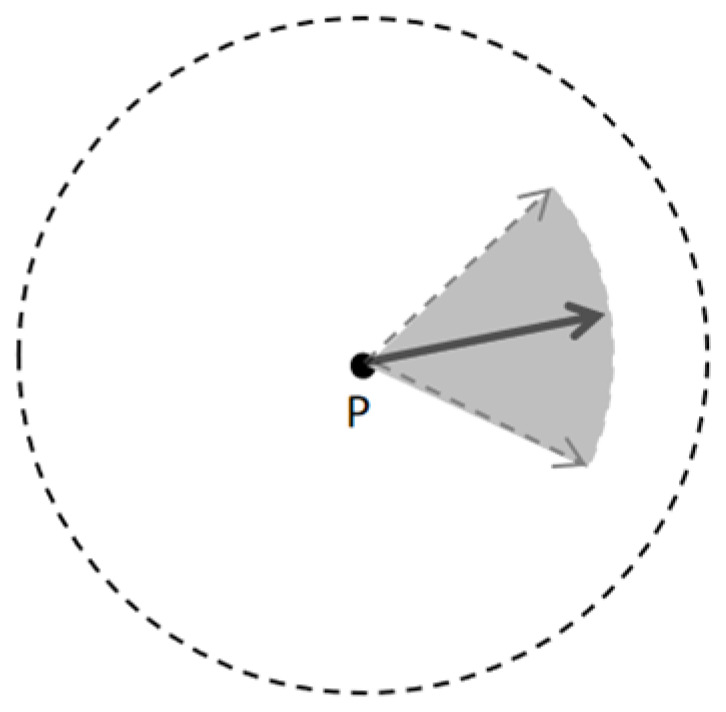
Schematic of new arrival condition.

In addition, the node extension of the traditional HybridA* algorithm needs to ensure that the position (x, y) and direction θ of the node are exactly the same as the target point when determining whether it reaches the target point or not, and this strict criterion is prone to triggering a large number of ineffective extensions when approaching the end point, which increases the computational overhead. This study addresses this problem by proposing a more flexible arrival condition setting, as shown in Figure 7: for each guidance point P, a circular region based on (x, y) and a sector region based on θ are set. A new node is considered to have reached the guidance point when it simultaneously satisfies that it is located within the circular region and the heading angle is within the fan-shaped interval.

**Figure 8:**
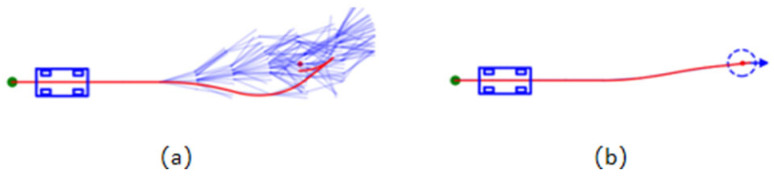
Comparison of node extensions before and after improvement: (**a**) Before improvement; (**b**) After improvement. A small green dot is the starting point, and a small orange dot is the target point. The blue line is the extension line, and the orange line is the path.

This flexible improvement reduces the problem of “hard-to-reach ends” and improves the overall search efficiency, and a comparison of the node extensions before and after the change is shown in Figure 8.

**Figure 9:**
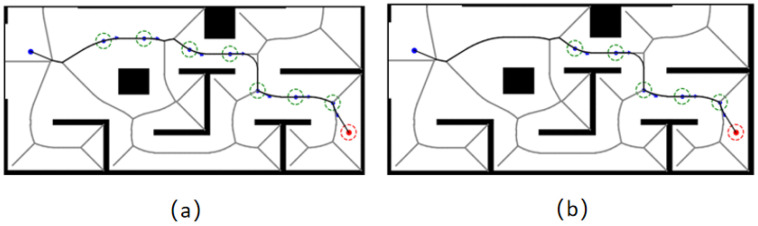
Schematic diagram of guide point selection: (**a**) Sampled guide point; (**b**) Filtered guide point. The small blue dot is the starting point, and the small orange dot is the target point. The green circle indicates the reachable range of the intermediate target points.

The forward guidance strategy proposed in this chapter provides a stage-like target point for the algorithm to search, and the Voronoi path and guidance point selection process are shown in Figure 9.

## 4. Safe Corridor Strategy

In order to more effectively control the amount of node search and ensure the safety of the generated paths, this paper goes on to propose an innovative safe corridor strategy, the implementation of which consists of two phases: corridor generation and application.

### 4.1. Corridor Generation

**Figure 10:**
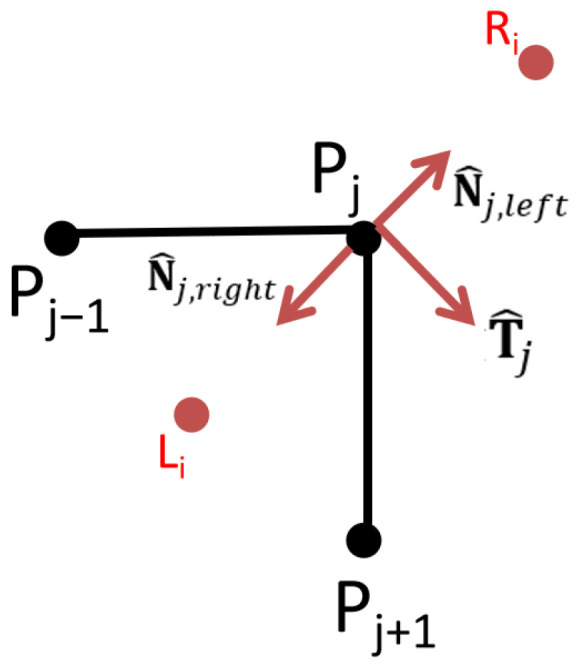
Calculation of corridor points.

The set of Voronoi path points generated in the previous section is denoted as $P=\left\{P_{1},P_{2},\ldots ,P_{n}\right\}$. For each Voronoi path point $P_{i}\left(x_{i},y_{i}\right)$, the corridor points $L_{i}$ and $R_{i}$ on both sides of the path point are computed, and the computation schematic is shown in Figure 10, which is firstly generated as the direction vector $T_{i}$ of the vectors $P_{i-1}$ and $P_{i+1}$, and the unit vector of $T_{i}$ is denoted as ${\hat{T}}_{i}$, and the unit normal vector of the corresponding two sides is ${\hat{N}}_{i,left}$ and ${\hat{N}}_{i,right}$. Then half of the predefined width is extended from the point $P_{i}$ in the direction of ${\hat{N}}_{i,left}$ and ${\hat{N}}_{i,right}$, that is to obtain the corridor points on both sides, as shown in Equations (10) and (11).

(10) $$L_{i}=P_{i}+\frac{D}{2}\cdot {\hat{N}}_{I,left}$$

(11) $$R_{i}=P_{i}+\frac{D}{2}\cdot {\hat{N}}_{i,right}$$

**Figure 11:**
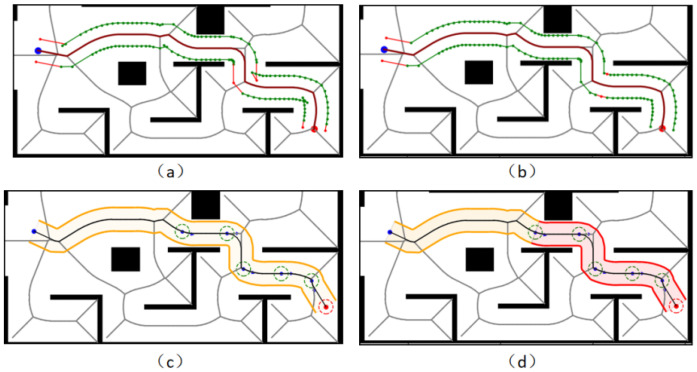
Corridor generation and processing: (**a**) Boundary generation; (**b**) Boundary processing; (**c**) Corridor generation; (**d**) Corridor delineation. The blue dot is the starting point, and the orange dot is the target point. The red areas along the corridor boundary indicate locations where self-intersections need to be resolved.

After calculating the corridor path points corresponding to all Voronoi path points, they are concatenated to form a corridor boundary line as shown in Figure 11a.

In places where the path curvature is large, the corridor points generated by neighboring path points will be misplaced, which leads to self-crossing of the corresponding corridor boundary here, as shown in Figure 11b. To solve this problem, this paper replaces the original corridor points with the intersection points, which effectively fixes the self-crossing of the corridor boundary and ensures the validity and consistency of the corridor structure, and generates corridors as shown in Figure 11c.

In addition, this paper divides the corridor into orange corridor and red corridor based on the distribution density of the guidance points, as shown in Figure 11d, the area with low distribution density means that the section of the path is farther away from the surrounding obstacles, which is recorded as the orange corridor portion, and the area with high distribution density means that the section of the path is closer to the surrounding obstacles, which is recorded as the red corridor portion.

### 4.2. Corridor Application

The generated corridor will constrain the subsequent HybridA* search scope, and any node that expands beyond the corridor boundary will be judged as an invalid node and will not participate in the subsequent expansion. This mechanism effectively ensures that the paths generated by the HybridA* algorithm are strictly located inside the preset safe corridors, which not only significantly reduces the expansion of invalid nodes, but also effectively guarantees the safety of the paths.

The segmented corridors will be used as the basis for the HybridA* dynamic step-size strategy. When the nodes are located in the orange corridor section with higher safety, large step-sizes are set for the algorithm for forward expansion, aiming to satisfy the need for fast path exploration. On the contrary, in the less safe corridor region, small step-sizes are set for the algorithm for forward expansion to ensure that path granularity and safety are the primary considerations. Such a dynamic step-size strategy enables HybridA* to flexibly cope with different environmental features and realize efficient path planning under the premise of safety. Especially in complex and changing environments, the algorithm’s adaptability and performance are enhanced by reasonably deploying the step-size, which allows for fast forward search in spacious areas as well as detailed exploration of optimal paths in narrow lots.

Additionally, we redefined the generation rules for the heuristic map based on the generated corridors to reduce computational time.

The heuristic map is a two-dimensional array storing distance statistic from the target point to all locations on the map, guiding the selection of optimal nodes during the algorithm’s search process. In multi-target point algorithms, heuristic map information is computed for each target point, leading to increased algorithm runtime.

**Figure 12:**
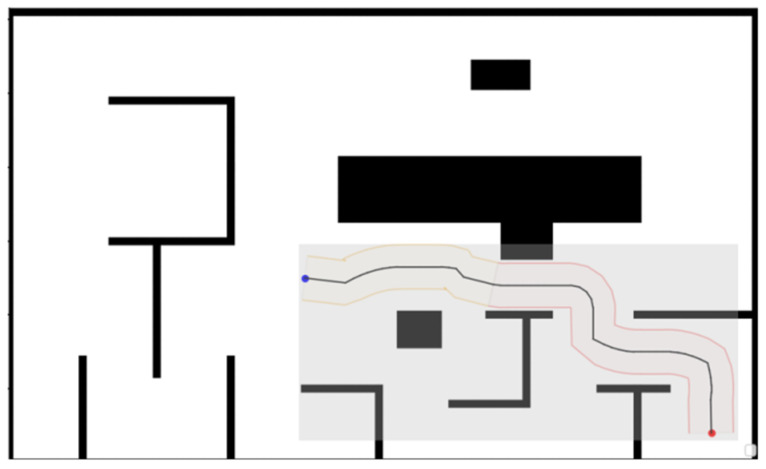
Schematic Diagram of Heuristic Information Computation. The dark gray areas represent regions of the heuristic map that have been recalculated.

In the algorithm proposed in this paper, since the Voronoi path provides the initial route and the safety corridor restricts the search space, the algorithm only requires heuristic information for positions within the corridor. Consequently, our original global map computation is reduced to computing a small rectangular map defined by the corridor’s shape and position, as shown in the gray area of Figure 12. During computation, only heuristic information for positions within the corridor is retained. Following this adaptation, the generated paths remain unchanged, but the computational time is significantly reduced.

## 5. GCHybridA*

**Figure 13:**
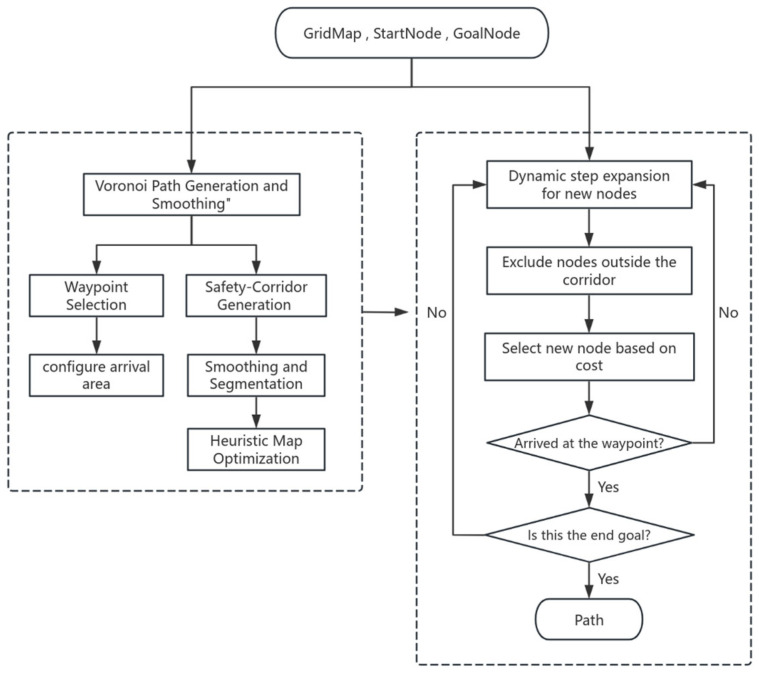
Flowchart of the GCHybridA* Algorithm.

The general workflow of the GCHybridA* algorithm is illustrated in Figure 13. The process begins by constructing a Voronoi diagram based on obstacle distribution in the grid map, followed by planning a Voronoi path between the start and target points, which is then smoothed. The smoothed path serves two primary purposes: first, it generates key guidance points through a screening strategy while configuring corresponding arrival regions; second, it is used to construct corresponding safety corridors that undergo intersection handling and segmentation; Finally, based on the corridor’s location and shape, perform small-scale heuristic map calculations to provide information for selecting the direction of path search.

These guidance points and safety corridors are integrated into the node exploration process: starting from the initial point, the algorithm performs adaptive step-size node expansion, retaining only nodes located within the corridors. Each node is evaluated using a cost function, and the node with the lowest cost is selected for further expansion. When a newly expanded node reaches the region of the current guidance point, the algorithm terminates and outputs the optimal path if this point is the final target; otherwise, it continues exploration toward the next guidance point.

## 6. Experimentation and Analysis

All experiments were conducted on a laptop equipped with an Intel(R) Core(TM) i5-8250U CPU @ 1.60 GHz and 16 GB of RAM. The algorithm was implemented in Python3.8 using PyCharm2023.2.4 as the integrated development environment. Key Python libraries utilized for geometric computation, visualization, and algorithm implementation include:

scipy.spatial.Voronoi for Voronoi diagram generation,

shapely.geometry for geometric operations and collision detection,

heapdict for efficient priority queue management in path search,

matplotlib for visualization of paths and experimental results.

These tools collectively support the efficient execution and evaluation of the proposed GCHybridA* algorithm.

### 6.1. Parameters and Map Settings

**Figure 14:**
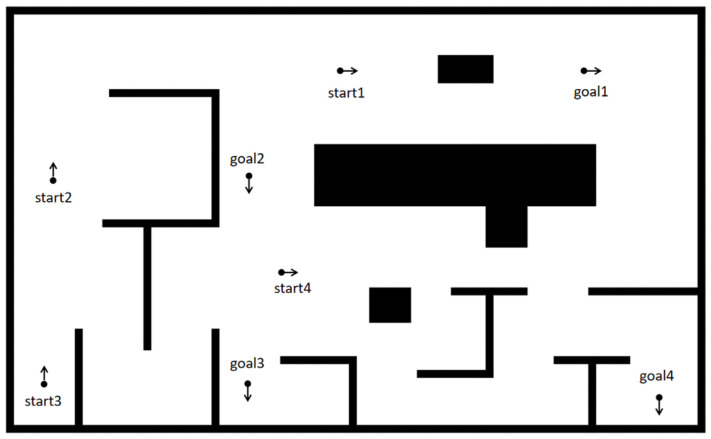
Map design.

The GCHybridA* algorithm proposed in this paper combines and applies the forward guidance strategy and the safe corridor strategy to the path search process of the HybridA* algorithm. In order to validate the effectiveness of the algorithm, we designed two parts, the comparison experiment and the ablation experiment, to study the performance of the algorithm under different obstacle distributions through four representative scenarios, as shown in Figure 14.

We evaluate the algorithm’s performance by calculating the number of node expansions, the number of potential collision points, and the computational time: The number of node expansions and the computational time both reflect the algorithm’s computational overhead. A reduction in this overhead directly correlates with increased search efficiency; potential collision points reflect the path safety, the locations where collisions with obstacles may occur due to control errors or environmental uncertainties in the actual execution. It is defined that when the path distance to an obstacle is ≤2 m (half of the vehicle width plus 0.5 m), the path is considered to have generated a potential collision point at that obstacle location, which is visualized as a yellow dot in the experimental diagrams.

The key hyperparameters in this paper (such as r, D, θ_threshold, etc.) were determined through the following approaches:

Guidance point arrival condition parameters (r and θ):

These parameters determine whether the current node has reached a guidance point. Excessively small values may cause “oscillatory” phenomena near the target point, making it difficult to reach the final destination. Conversely, overly large values may lead to node pose deviation, resulting in degraded path quality (e.g., close proximity to obstacles or jagged paths). Through experimental comparison, we ultimately adopted r = 1 m and θ = 30° combination, which yielded the ideal results presented in the experiments.

Corridor width:

This parameter is determined through a dynamic adaptive generation method. The algorithm initially attempts larger corridor width values and then iteratively reduces the width if collisions with obstacles occur until an environment-appropriate width is achieved. As shown in the figures, the generated corridor widths vary across different maps, demonstrating the flexibility of this approach.

Dynamic step-size:

The short step-size is set to 1.5 m to match parameter r in arrival conditions, preventing oscillation near target points. The long step-size is 3 m, which appropriately increases search speed without compromising path quality (avoiding jagged paths).

All experiments are conducted under the parameters of Table 1.

### 6.2. Comparative Experiments

The HybridA* algorithm is usually combined with the Reed-shepps curve to reduce the amount of computational scaling, which is noted as HybridA*_RS algorithm [14], and the comparison experiment compares the GCHybridA* algorithm with both the HybridA* algorithm and the HybridA*_RS algorithm to validate the effectiveness of this paper’s algorithm compared to the original algorithm.

**Figure 15:**
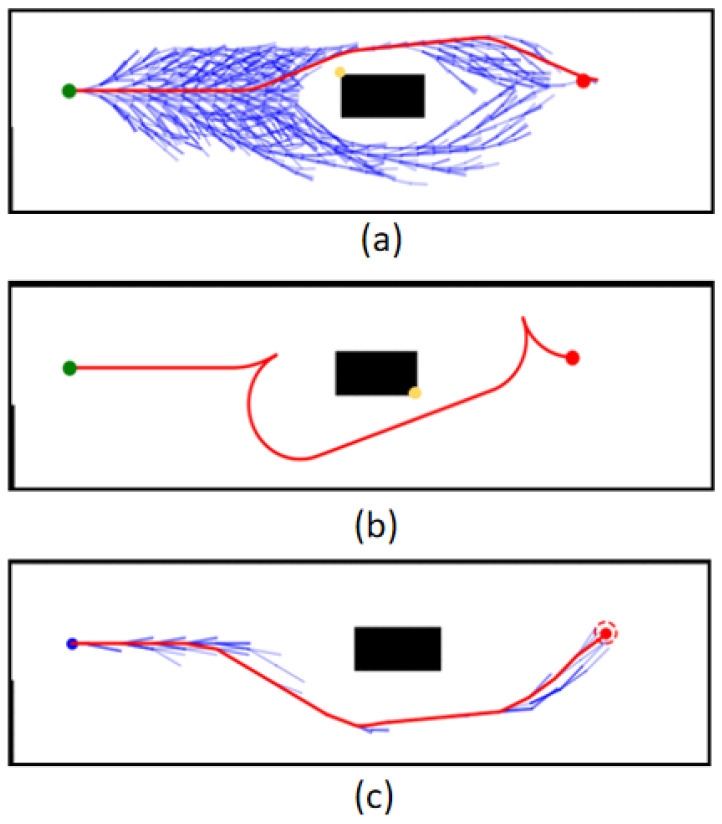
Comparative experiments of map 1: (**a**) HybridA*; (**b**) HybridA*_RS (**c**) GCHybridA*. The green dot indicates the starting point, the orange dot indicates the endpoint. The yellow dot indicates a potential collision risk point.

Map 1 shows the case of bypassing an obstacle, under which: the HybridA* algorithm shows more node expansions and there is a potential collision point, as shown in Figure 15a; the HybridA*_RS algorithm generates a very low-quality path and there is a potential collision point, as shown in Figure 15b; and the GCHybridA* algorithm accomplishes the bypassing with a low amount of node expansions task and does not generate any collision risk, as shown in Figure 15c.

**Figure 16:**
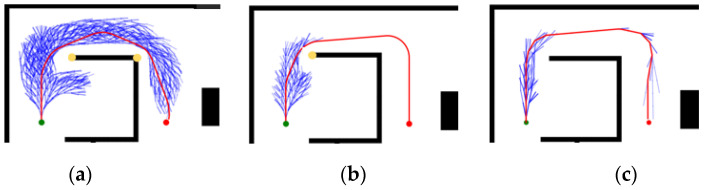
Comparative experiments of map 2: (**a**) HybridA*; (**b**) HybridA*_RS; (**c**) GCHybridA*. The green dot indicates the starting point, the orange dot indicates the endpoint. The yellow dot indicates a potential collision risk point.

Map 2 shows the case of moving around an obstacle, under which: the HybridA* algorithm shows more node expansion and the path generates two potential collision points, as shown in Figure 16a; the HybridA*_RS algorithm has less node expansion but the path also generates one potential collision point, as shown in Figure 16b; the GCHybridA* algorithm has a node expansion amount close to that of the HybridA*_RS, but the path has better safety performance and does not produce any potential collision point, as shown in Figure 16c.

**Figure 17:**
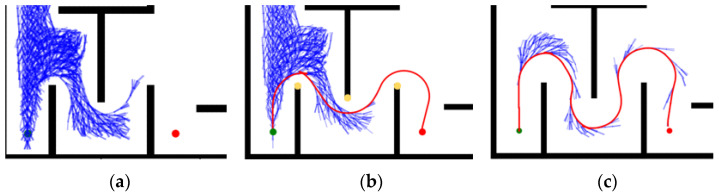
Comparative experiments of map 3: (**a**) HybridA*; (**b**) HybridA*_RS; (**c**) GCHybridA*. The green dot indicates the starting point, the orange dot indicates the endpoint. The yellow dot indicates a potential collision risk point.

Map 3 shows the case of going through a continuous u-turn, under this map: the HybridA* algorithm fails to search the path even though the node search amount exceeds 4000, which is considered as a search failure, as shown in Figure 17a; the HybridA*_RS algorithm searches the path after a large amount of ineffective extensions, and there are three potential collision points in the path, as shown in Figure 17b; The GCHybridA* algorithm has the smallest amount of expansion and the planned path is safer with no potential collision points, as shown in Figure 17c.

**Figure 18:**
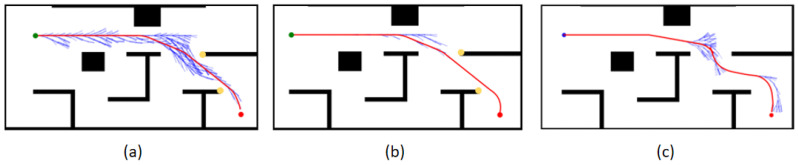
Comparative experiments of map 4: (**a**) HybridA*; (**b**) HybridA*_RS; (**c**) GCHybridA*. The green dot indicates the starting point, the orange dot indicates the endpoint. The yellow dot indicates a potential collision risk point.

Map 4 shows the case of traversing complex terrain, under which: the three algorithms have similar node extensions, but in terms of safety performance, both the HybridA* algorithm and the HybridA*_RS algorithm generate two potential collision points, as shown in Figure 18a,b; and the GCHybridA* algorithm generates paths that always keep a safe distance from obstacles, with no potential collision points generated, as shown in Figure 18c.

Taken together, the GCHybridA* algorithm performs well in terms of both safety and computation, significantly outperforming other methods in the four maps of the comparison experiment. This indicates that the GCHybridA* algorithm proposed in this paper not only significantly improves the safety of path planning but also maintains a high computational efficiency.

### 6.3. Ablation Experiments

The method that applies only the forward guidance strategy to the path search process of the HybridA* algorithm is denoted as the GHybridA* algorithm, while the method that applies only the safe corridor strategy to the path search process of the HybridA* algorithm is denoted as CHybridA*. The ablation experiments compare the performance of the GHybridA* algorithm, the CHybridA* algorithm, and the GCHybridA* algorithm under four maps to verify the effectiveness of the combined application of the two strategies.

**Figure 19:**
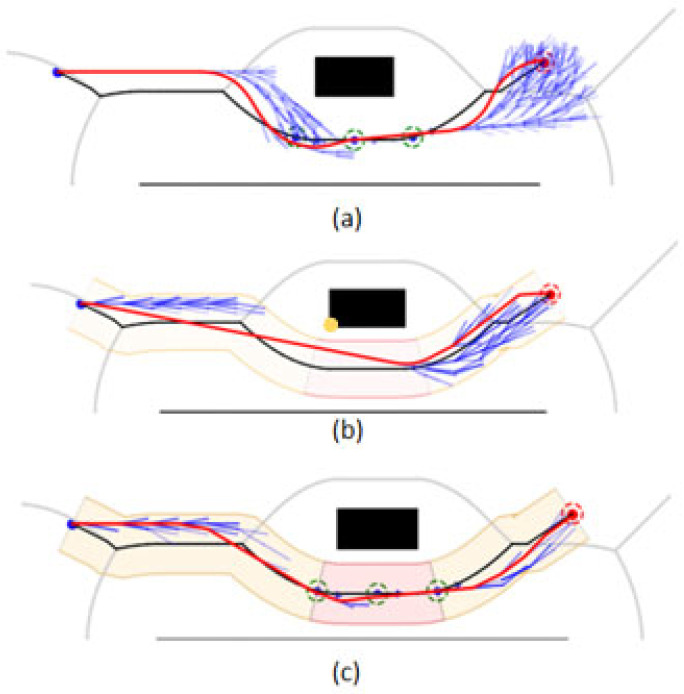
Ablation experiments of map 1: (**a**) GHybridA*; (**b**) CHybridA*; (**c**) GCHybridA*. The blue dot marks the starting point, the orange dot the endpoint. The yellow dot indicates a potential collision risk point. The black line represents the initial path, the blue line the extended path, and the red line the final path.

Under Map 1: the paths of both the GHybridA* and GCHybridA* algorithms maintain a sufficiently safe distance from obstacles, but the GHybridA* algorithm has more node extensions, as shown in Figure 19a,c; and the paths planned by the CHybridA* algorithm have a potential collision point, which lacks sufficient safety, as shown in Figure 19b.

**Figure 20:**
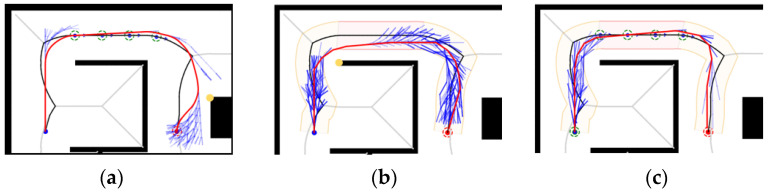
Ablation experiments of map 2: (**a**) GHybridA*; (**b**) CHybridA*; (**c**) GCHybridA*. The blue dot marks the starting point, the orange dot the endpoint. The yellow dot indicates a potential collision risk point. The black line represents the initial path, the blue line the extended path, and the red line the final path.

Under Map 2: the three algorithms’ node extensions are close to each other, but both the GHybridA* algorithm and the CHybridA* algorithm generate a potential collision point, as shown in Figure 20a,b; while the GCHybridA* algorithm does not have any potential collision point on the left or right side, and it always maintains a safe distance from the obstacles, as shown in Figure 20c.

**Figure 21:**
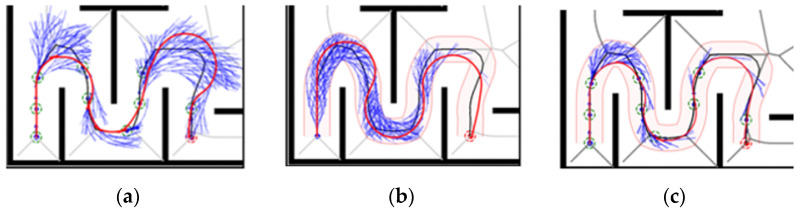
Ablation experiments of map 3: (**a**) GHybridA*; (**b**) CHybridA*; (**c**) GCHybridA*. The blue dot marks the starting point, the orange dot the endpoint. The yellow dot indicates a potential collision risk point. The black line represents the initial path, the blue line the extended path, and the red line the final path.

Under Map 3: All three algorithms have better safety. However, the GHybridA* algorithm has a large number of invalid node expansions due to the lack of corridor search constraints, as shown in Figure 21a; the CHybridA* algorithm also suffers from a high number of node expansions, as shown in Figure 21b; and the GCHybridA* algorithm has the smallest amount of node expansions, as shown in Figure 21c.

**Figure 22:**
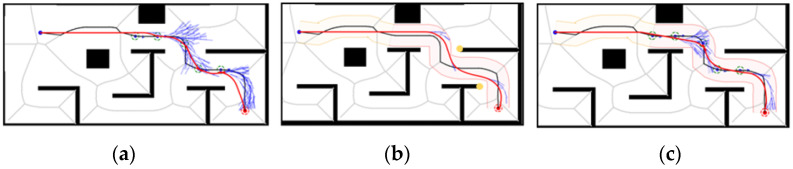
Ablation experiments of map 4: (**a**) GHybridA*; (**b**) CHybridA*; (**c**) GCHybridA*. The blue dot marks the starting point, the orange dot the endpoint. The yellow dot indicates a potential collision risk point. The black line represents the initial path, the blue line the extended path, and the red line the final path.

Under Map 4: the GHybridA* algorithm has no potential collision point generation, as shown in Figure 22a; the CHybridA* algorithm has the least amount of node expansion but generates two potential collision points, as shown in Figure 22b; the GCHybridA* algorithm has the same good safety as the GHybridA* algorithm but with less node expansion than the CHybridA* algorithm is less, as shown in Figure 22c.

Taken together, the GCHybridA* algorithm has the best safety among the four maps in the ablation experiments, and its performance in terms of computational volume is also very stable, always with a small amount of node expansion, which fully demonstrates the effectiveness of the combination of forward guidance and safe corridor strategies for the path planning of the GCHybridA* algorithm.

## 7. Experimental Analysis

Table 2 provides a detailed summary of the performance of the five algorithms across four different maps, comprehensively presenting data on three key metrics: number of potential collision points, node expansion count and path generation time.

**Figure 23:**
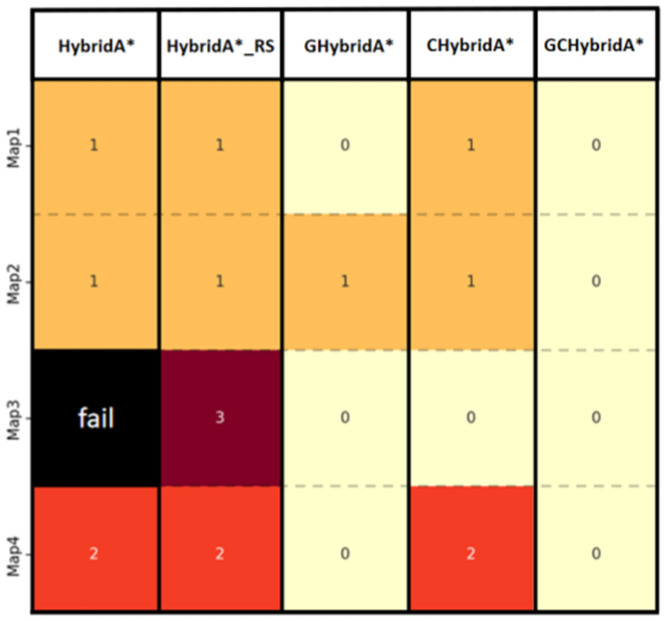
Heat map of potential collision point contrasts of each method. The number represents the collision point count for the current algorithm on the current map. Light colors indicate low collision risk, while dark colors indicate high collision risk.

In terms of safety, the paths generated by the GCHybridA* algorithm do not generate potential collision points on the four different maps, fully demonstrating its stable safety, as shown in Figure 23.

**Figure 24:**
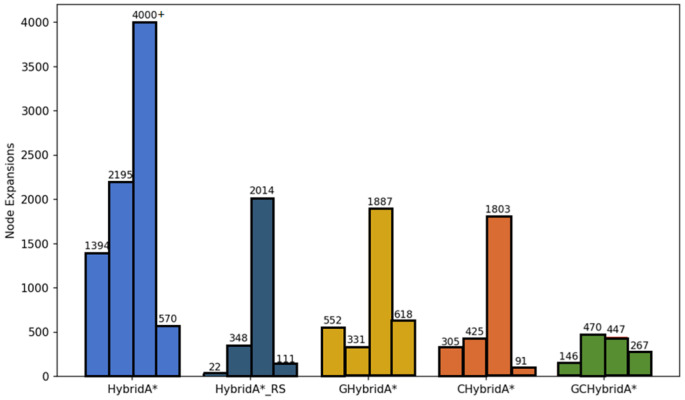
Bar chart comparing the node expansion of each method.

Regarding node expansion, as shown in Table 3, the GCHybridA algorithm demonstrates lower total node count, mean value, and variance compared to other algorithms. This indicates that the proposed algorithm maintains the most stable performance across different maps, consistently operating at a lower level, as Figure 24 shown.

**Figure 25:**
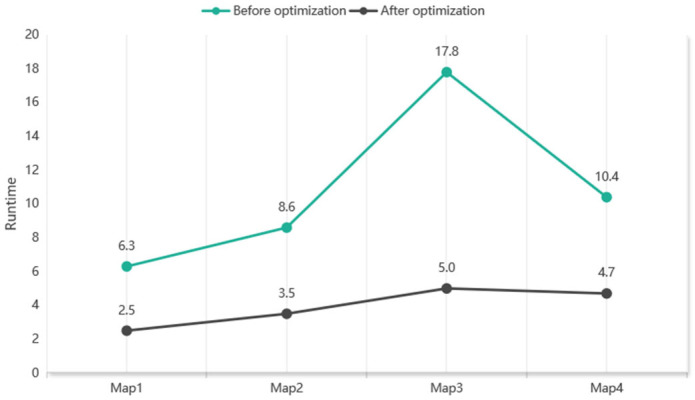
Comparison of GCHybridA* Runtimes (Original vs. Optimized).

In terms of algorithmic time performance, the new heuristic computation strategy significantly reduces the computational time of the GCHybridA* algorithm, demonstrating particularly notable improvements on map 3 with the highest number of target points, the comparison before and after the improvement is shown in Figure 25.

**Figure 26:**
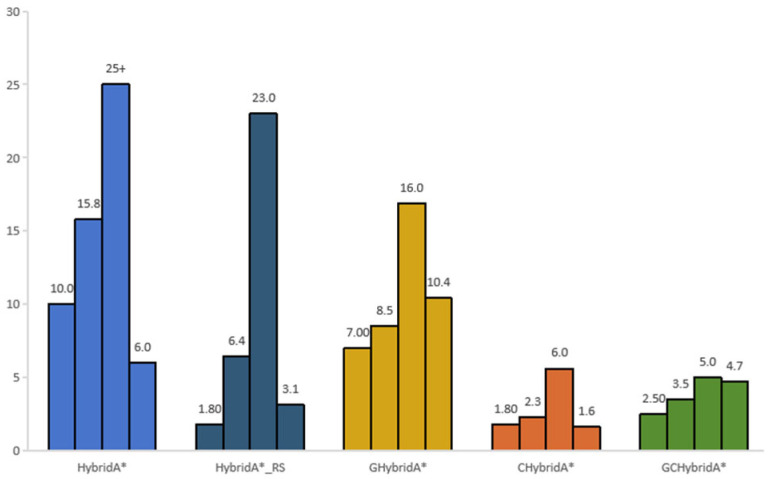
Bar chart comparing the path generation time of each method.

The runtime performance of the five algorithms across four maps is shown in Figure 26. The CHybridA* and GCHybridA* algorithms significantly outperform the other three in terms of total duration, average runtime, and variance. This superiority stems from their reduced node expansion counts and the application of corridor-based heuristic map computation rules. The computational time of the CHybridA* algorithm is slightly lower than that of the GCHybridA* algorithm because it does not employ multi-objective guidance, and its heuristic map is computed only once. Even so, the temporal performance of the GCHybridA* algorithm remains close to that of the CHybridA*.

This paper designs an experiment to conduct sensitivity analysis on key parameters.

1. For the two parameters of the arrival area—GOAL_AREA(1.0 m) and HEADING_SECTOR(15°)—we designed two sets of new parameter configurations and compiled experimental data across all maps.

Stricter arrival area configuration: GOAL_AREA(0.8 m), HEADING_SECTOR(10°). Compared to the original configuration, this setup resulted in a total node expansion increase of 973 across the four maps, a 9.4-s increase in total algorithm runtime, and no increase in potential collision points. The decrease in time efficiency primarily stems from sensitivity to matching condition changes at guide points in narrow turning areas of certain maps, requiring more time to find suitable paths.

Relaxed Goal Area Configuration: GOAL_AREA(1.2 m), HEADING_SECTOR(20°). Compared to the original parameter configuration, this setup reduces the total node expansion across the four maps by 45 nodes and decreases the total runtime by approximately 1.5 s, with no increase in potential collision points. Computational efficiency shows only minor differences from the original configuration, as the primary computational overhead remains concentrated in heuristic map processing when arrival conditions are relaxed. However, the enlarged heading angle sector causes oscillatory behavior in narrow path segments.

2. For the dynamic step size parameter STEP-SIZE (0.3/3.0 m), we designed two new parameter configurations and collected experimental data across four maps.

Increased step size configuration (0.4/3.5 m): Compared to the original configuration, this setup increased total node expansion by 302 nodes across the four maps, extended total algorithm runtime by 2.55 s, and did not increase potential collision points. The time increase resulted from overly large step sizes causing the matching region to cross guide points.

Shorter step size configuration (0.2/2.5 m): Compared to the original configuration, this setup increased total node expansion by 897 across the four maps, extended total algorithm runtime by 5.85 s, and showed no increase in potential collision points. The time increase stemmed from smaller step sizes requiring more computations to reach target points at the same distance.

In summary, node expansion and runtime are highly sensitive to stricter arrival area requirements and stride parameter changes, while safety remains stable due to dual safeguards from the Vino path and safety corridor.

Discussion on algorithm completeness and bounded computational complexity:

The method employed here achieves resolution completeness within corridors, meaning it can find a path when resolution is sufficiently high and a path satisfying kinematic constraints exists within the corridor. Corridor-based methods often overlook other feasible or superior solutions outside the corridor. This paper addresses this by calculating a shortest initial path as the corridor’s foundation, where the optimality of the initial path indirectly ensures subsequent path quality. However, when multiple initial Vino paths have similar lengths, the algorithm may miss a corridor environment that is better overall.

Regarding bounded suboptimality, GCHybridA* inherits the characteristics of the original HybridA* algorithm and does not exhibit strict bounded suboptimality. Furthermore, this algorithm aims to balance three sets of metrics—safety, path quality, and computational efficiency—while ensuring the path’s kinematic feasibility. This further complicates the proof of strict bounded suboptimality.

In summary, the GCHybridA* algorithm significantly reduces the search space and provides higher-quality paths compared to the original algorithm. Experimental results demonstrate its outstanding performance in safety, efficiency, and robustness, offering an effective solution for autonomous vehicle path planning.

## 8. Discussion

The proposed GCHybridA* algorithm improves three major shortcomings of traditional hybrid A* algorithms in complex, unstructured environments: inefficient search, unstable performance, and insufficient path safety. These enhancements make GCHybridA* a robust and efficient solution for global path planning in static environments, providing reliable, safe, and kinematically feasible reference paths for autonomous navigation systems.

The positive outcomes achieved in static environments lay a solid foundation for future integration into a comprehensive autonomous driving technology stack. Subsequent logical steps include:

System Integration: Embedding GCHybridA* into integrated frameworks like Autoware as a global planner, evaluating its interoperability with standardized localization, perception, and local planning modules.

Real-world evaluation: Conduct research within the integrated system using simulations (e.g., CARLA or Gazebo) to assess how global path quality and stability (e.g., smoothness, safe path width, and replanning frequency) impact the overall performance of downstream local planners in dynamic scenarios. Transition from simulation environments to limited real-world testing in controlled settings to validate the algorithm’s readiness for practical deployment.

## 9. Patents

A patent has been granted for the methodology presented in this work. The details are as follows: Patent No. ZL202510486514.0, “一种基于引导走廊的HybridA*路径规划方法” (A HybridA* Path Planning Method Based on a Guiding Corridor), granted by the China National Intellectual Property Administration to Changchun University.

## Figures and Tables

**Table 1:** Experimental parameter settings.

Parameters	Value	Description
XY_RESO	1.0 [m]	The resolution in the x-y plane for discretizing the space.
YAW_RESO	15 [degree]	The angular resolution for discretizing yaw angles.
STEP-SIZE	0.3/3.0 [m]	adaptive step-size node expansion
N_STEER	3.0	The number of discrete steering commands available.
G_Weight	5.0	Weight factor for the actual cost (G-cost) in the HybridA* algorithm.
H_Weight	5.0	Weight factor for the heuristic cost (H-cost) in the HybridA* algorithm.
RF	4.5 [m]	Distance from the rear of the vehicle to its front end.
RB	1.0 [m]	Distance from the rear of the vehicle to its back end.
W	3.0 [m]	Width of the vehicle.
WD	0.7 × W [m]	Distance between the left and right wheels.
WB	3.5 [m]	Distance between the front and rear axles of the vehicle.
MAX_STEER	0.6 [rad]	Maximum possible steering angle for the vehicle.
GOAL_AREA	1.0	Threshold distance within which a node is considered to have reached the goal.
HEADING_SECTOR	15 [degree]	the allowable heading deviation range of the node relative to the guidance point
INITIAL_CORRIDOR_WIDTH	6 [m]	The initial safety corridor width is set to 6 m. If a collision with an obstacle is detected, it automatically reduces by 1 m.

**Table 2:** Comparative results of the methods.

Evaluation	Number of Collision Points per Map				Number of Node Expansions per Map				Path Generation Time per Map			
Map Method	Map 1	Map 2	Map 3	Map 4	Map 1	Map 2	Map 3	Map 4	Map 1	Map 2	Map 3	Map 4
HybridA*	1	1	Fail	2	1394	2195	Fail	570	9.97	15.78	Fail	6.00
HybridA*_RS	1	1	3	2	22	348	2014	111	1.79	6.41	23.02	3.11
GHybridA*	0	1	0	0	552	331	1887	618	7.03	8.50	16.88	10.36
CHybridA*	1	1	0	2	305	425	1803	91	1.75	2.89	5.56	1.59
GCHybridA*	0	0	0	0	146	470	447	267	2.46	3.45	5.05	4.73

**Table 3:** Node expansion volume analysis table.

Method	Total	Mean	Variance
HybridA*	8159	2039.75	1,610,960.44
HybridA*_RS	2495	623.75	658,462.19
GHybridA*	3388	847.00	371,830.50
CHybridA*	2624	656.00	452,849.00
GCHybridA*	1330	332.50	17,772.25

## Data Availability

The original contributions presented in this study are included in the article. Further inquiries can be directed to the corresponding author.

## References

[1] Abdallaoui S., Aglzim E.H., Chaibet A., Kribèche A. (2022). Thorough review analysis of safe control of autonomous vehicles: Path planning and navigation techniques. Energies.

[2] Chen G., Luo N., Liu D., Zhao Z., Liang C. (2021). Path planning for manipulators based on an improved probabilistic roadmap method. Robot. Comput.-Integr. Manuf..

[3] Li Y., Xu D., Zhou C. (2020). Cooperation path planning of dual-robot based on self-adaptive stepsize RRT. Robot.

[4] Yang Z., Wang Y., Qi A. (2019). Improved RRT* algorithm based global obstacle avoidance planning for unmanned surface vehicles. Ship Sci. Technol..

[5] Li Y., Wei W., Gao Y., Wang D., Fan Z. (2020). PQ-RRT*: An improved path planning algorithm for mobile robots. Expert Syst. Appl..

[6] Le A.V., Prabakaran V., Sivanantham V., Mohan R.E. (2018). Modified A-star algorithm for efficient coverage path planning in Tetris inspired self-reconfigurable robot with integrated laser sensor. Sensors.

[7] Martins O.O., Adekunle A.A., Olaniyan O.M., Bolaji B.O. (2022). An Improved multiobjective a-star algorithm for path planning in a large workspace: Design, Implementation, and Evaluation. Sci. Afr..

[8] Wang X., Zhang H., Liu S., Wang J., Wang Y., Shangguan D. (2022). Path planning of scenic spots based on improved A* algorithm. Sci. Rep..

[9] Ravankar A., Ravankar A.A., Kobayashi Y., Hoshino Y., Peng C.C. (2018). Path smoothing techniques in robot navigation: State-of-the-art, current and future challenges. Sensors.

[10] Zhan W., Chen J., Chan C.-Y., Liu C., Tomizuka M. Spatially-partitioned environmental representation and planning architecture for on-road autonomous driving. Proceedings of the 2017 IEEE Intelligent Vehicles Symposium (IV).

[11] Gutjahr B., Gröll L., Werling M. (2017). Lateral vehicle trajectory optimization using constrained linear time-varying MPC. IEEE Trans. Intell. Transp. Syst..

[12] Zhou X., Zhu J., Zhou H., Xu C., Gao F. EGO-Swarm: A fully autonomous and decentralized quadrotor swarm system in cluttered environments. Proceedings of the 2021 IEEE International Conference on Robotics and Automation (ICRA).

[13] Mane S.B., Vhanale S. (2019). Genetic algorithm approach for obstacle avoidance and path optimization of mobile robot. Adv. Intell. Syst. Comput..

[14] Dolgov D., Thrun S., Montemerlo M., Diebel J. (2010). Path planning for autonomous vehicles in unknown semi-structured environments. Int. J. Robot. Res..

[15] Tang K., Hirota X., Wu Y., Dai Y., Jia Z. (2021). Path planning based on improved HybridA* algorithm. J. Adv. Comput. Intell. Intell. Inform..

[16] Chi Z., Yu Z., Wei Q., He Q., Li G., Ding S. (2023). High-efficiency navigation of nonholonomic mobile robots based on improved HybridA* algorithm. Appl. Sci..

[17] Dang C.V., Ahn H., Lee D.S., Lee S.C. (2022). Improved analytic expansions in hybrid A-star path planning for non-holonomic robots. Appl. Sci..

[18] Chang T., Tian G. (2024). Hybrid A-star path planning method based on hierarchical clustering and trichotomy. Appl. Sci..

[19] Meng T., Yang T., Huang J., Jin W., Zhang W., Jia Y., Wan K., Xiao G., Yang D., Zhong Z. (2023). Improved hybrid A-star algorithm for path planning in autonomous parking system based on multi-stage dynamic optimization. Int. J. Automot. Technol..

[20] Lian J., Ren W., Yang D., Li L., Yu F. (2023). Trajectory planning for autonomous valet parking in narrow environments with enhanced HybridA* search and nonlinear optimization. IEEE Trans. Intell. Veh..

[21] Sedighi S., Nguyen D.-V., Kuhnert K.-D. Guided hybrid A-star path planning algorithm for valet parking applications. Proceedings of the 2019 5th International Conference on Control, Automation and Robotics (ICCAR).

[22] Sedighi S., Nguyen D.-V., Kapsalas P., Kuhnert K.-D. Implementing Voronoi-based guided HybridA* in global path planning for autonomous vehicles. Proceedings of the 2019 IEEE Intelligent Transportation Systems Conference (ITSC).

[23] Cui G., Yin Y., Xu Q., Song C., Li G., Li S. (2024). Efficient path planning for automated valet parking: Integrating HybridA* search with geometric curves. Int. J. Automot. Technol..

[24] Qin Z., Chen X., Hu M., Chen L., Fan J. (2020). A novel path planning methodology for automated valet parking based on directional graph search and geometry curve. Robot. Auton. Syst..

